# Mechanisms involved in altered bone metabolism in diabetes: a narrative review

**DOI:** 10.1186/s40200-016-0275-1

**Published:** 2016-11-15

**Authors:** Maryam Ghodsi, Bagher larijani, Abbass Ali Keshtkar, Ensieh Nasli-Esfahani, Sudabeh Alatab, Mohammad Reza Mohajeri-Tehrani

**Affiliations:** 1 Diabetes Research Center (DRC), Endocrinology and Metabolism Research Institute (EMRI), Tehran University of Medical Sciences (TUMS), Tehran, Iran; 2 Endocrinology and Metabolism Research Center (EMRC), Endocrinology and Metabolism Resarch Institute (EMRI), Tehran University of Medical Sciences (TUMS), Tehran, Iran; 3 Department of Health Sciences Education Development, School of Public Health (SPH), Tehran University of Medical Sciences (TUMS), Tehran, Iran; 4 Urology Research Center (URC), Tehran University of Medical Sciences (TUMS), Tehran, Iran

**Keywords:** Diabetes, Osteoporosis, Vitamin D, Hyperglycemia, Insulin, IGF-1, Adiposity, Visfatin, Resistin, Leptin, Adiponectin, Inflammation, Vasculopathy, Antidiabetic Agents, Secretion

## Abstract

Many studies have shown that change in metabolism caused by diabetes can influence the bone metabolism in a way that quality and strength of bone is decreased. A 6 times and 2 times increased risk of fracture is reported in patients with type 1 and type 2 diabetes, respectively. There are several mechanisms by which diabetes can affect the bone. The fact that some of these mechanisms are acting in opposite ways opens the door for debate on pathways by which diabetes affects the bones. On the other hand, bone is not a simple organ that only get influence from other organs, but it is an endocrine organ that by secreting the agents such as osteocalcin, adiponectin and visfatin which can affect the insulin sensitivity and metabolism. In this paper we tried to briefly assess the latest finding in this matter.

## Background

Osteoporosis is a common skeletal system disease characterized by bone density attenuation and normal bone microstructure deterioration. All these changes can lead to an increased risk of fracture [1]. Many of these fractures are related to increased morbidity and mortality seen in these patients. It has been shown in recent years that diabetes, as a common disease with an increasing prevalence, is associated with increased morbidity and mortality. Moreover, recent data has revealed that both type 1 and 2 diabetes are associated with an increased risk of osteoporosis related fractures [2]. Diabetes-induced changes in metabolism significantly affect the bone metabolism and increase the risk of fracture by six times in type 1 and by 2 times in type 2 diabetic patients [3]. Although many studies have shown the negative effects of diabetes on the bone quality and strength, the debate is still open [4, 5].

Part of this discrepancy can be attributed to the mechanisms involved. There are several mechanisms by which diabetes can affect the bone (Table 1). Some of these mechanisms act in opposite directions. In this paper we tried to briefly review the mechanisms.

**Table 1:** Summary of main mechanisms by which diabetes affect bone

1- Hyperglycemia	• Differentiation of MSCs to fat cells↑, osteoblast function and number↓, osteoblast gene expression↓, osteoblast maturation↓ • AGEs↑, Oxidative stress↑, Adipogenesis↑
2- Insulin, IGF-1	Insulin has osteogenesis effects: • directly: osteoblast function and number↑, osteoblast maturation↑ • Indirectly: control of blood glucose levels, IGF-1, PTH,and vitamin D
3- Calcium, vitamin D and calciotrophic hormones	• Calcium loss (kidney and bones) ↑ • vitamin D deficiency (genetic polymorphisms)↑ • PTH ↓
4- Diabetic bone marrow adiposity	Osteopenic adiposity: • Differentiation of MSCs to fat cells↑ • Stimulate adipogenesis through PPARγ↑ • Inhibition of osteoblast profanation by FFAs
5- Secretion of bone marrow cells	• Adiponectin: ↑ inT1DM (micro vascular complications), ↑↓in T2DM • Leptin: ↓ inT1DM,↑in T2DM • Osteocalcin↓
6- inflammation	• Systemic inflammation (chronic) • Bone inflammation: inflammatory cells & mediators ↑, markers of osteoblast inhibition↑
7- Vasculopathy	• vascular supply to bone↓ • Fragility fracture risk↑
8- Antidiabetic agents	• positive effects on bone metabolism: Insulin, Metformin, Sulfonylurea, DPP-4 Inhibitors (Sitagliptin) • negative effects on bone metabolism: TZDs

*AGEs* Advanced Glycosylation End-Products, *IGF-1* Insulin, Insulin-like growth factor-1,*PTH* parathyroid hormone, *PPARγ* peroxisome proliferator-activated receptor γ, *FFAs* free fatty acids, *DPP-4* Dipeptidyl peptidase-4, *TZDs* thiazolidinediones

## Main text

1. Hyperglycemia - Hyperglycemia can attenuate the bone density through several mechanisms.
  1.1. Toxic effects caused by high levels of glucose could directly reduce the osteoblast function and number [6].
  1.2. High levels of glucose could independently change the levels of osteoblast gene expression through the osmotic and non-osmotic pathways [7]. These changes result in inhibition of osteoblast maturation and bone mineralization.
  1.3. Impairment of osteoblast maturation, caused by high glucose levels, results in an impaired response to 1, 25 hydroxy vitamin D3 (1, 25(OH)-D3). This indirectly causes the down regulation of vitamin D receptors.
  1.4. Production of glycation end products: High glucose levels, through non-enzymatic pathways, may induce glycation of various proteins and produce the products called advanced glycosylation end-products (AGEs). These products are seen in different tissues of diabetic subjects and are supposed to be involved in pathogenesis of diabetes [8]. It seems hyperglycemia and AGEs have a major role in fragility of bones in both type of diabetes [9]. In cortical bone, accumulation of AGEs causes an increase in production of cross-links between collagens. Although this process can increases the rigidity and hardness of collagen, it does not affect the bone mineralization. In fact there is a negative relation between AGEs and size and fragility of the human trabecular bone [10] which could explain the increased bone fragility and fracture in diabetic subjects. Moreover, apart from the direct effects of high glucose, accumulation of AGEs has a direct inhibitory effect on the proliferation and differentiation of bone cells.
  1.5. Oxidative stress: production and accumulation of AGEs can induce the cellular apoptosis through production of reactive oxygen species (ROS) and oxidative stress [11].
  1.6. High levels of glucose causes an increase in differentiation of bone marrow mesenchymal cells into adipocytes, which in turn could rise the adipogenesis and attenuate the osteogenesis [12].
2. Insulin, insulin like growth factor-1 (IGF-1) and other growth factors
  2.1. Apart from IGF-1, insulin has some osteogenic effects [13], and directly and indirectly induces the production and differentiation of osteoblasts [14–16]. The direct effect is mediated through insulin and IGF-1 receptors located on osteoblast. For example it has been shown that in response to exogenous insulin, the osteoblasts cultured cells increase collagen production [17]. The indirect effect of insulin is mediated through both the control of blood glucose levels and its effects on parathyroid hormone (PTH), IGF-1 and vitamin D [18–20]. In animal models of type 1 diabetes(T1DM), reduced bone density and osteoporosis has been reported [21, 22]. Moreover in clinical studies, it has been shown that bone mineral density(BMD) of femoral neck in T1DM subjects is lower than type 2 diabetes (T2DM) patients [23]. This finding is explained with lack of insulin in T1DM patients and could account for higher risk of osteopenia and osteoporosis in young age T1DMs [24]. Nevertheless higher BMD levels which have been reported in patients with T2DM in comparison with patients with T1DM could be explained by higher body weight levels and BMD in the T2DM patients [25].
  2.2. The insulin analogue, IGF-1, can affect the bone metabolism. In fact IGF-1 is regarded as a key regulator of bone metabolism that increases both the bone deposition in matrix and osteoblasts recruitment and decreases the bone loss and collagen destruction in the bone [26]. In spontaneously diabetic BB rats, the osteoblasts number is normal, however there is an impairment in bone mineralization which is similar to what is seen in osteomalacia (osteomalacia-like mineralization defect) [27]. Moreover, use of controlled release of IFG-1 as a drug model represented a promising results for bone defects that do not heal under normal therapeutic conditions [28]. In clinical studies, an association between vertebral fractures and decreased levels of IGF-1, has been reported in postmenopausal T2 diabetic women [15, 29]. The IGF-1 and 17-*β*-estradiol can be considered as the most significant hormonal determinants of the BMD of the hip and femoral neck in young men, however, the correlation do not remain significant for IGF-1 in men aged over 60 years [30]. On the other hand, IGF-1 levels are lower in patients with T1DM than patients with T2DM [31]. And last but not least, according to cross-sectional trials evaluated the association between bone turnover markers (BTMs) and fracture, IGF-1 might be considered as a predictor of fracture risk in diabetes [32]. Further investigations are highly recommended in order to provide comprehensive knowledge about related mechanisms.
3. Calcium, vitamin D and calciotrophic hormones

Calcium and its metabolism have a fundamental role in bone metabolism. Therefore any change that affects the equilibrium between systemic factors balancing the calcium levels can result in bone loss [33]. The simultaneous presence of vitamin D deficiency and diabetes (either type1 or 2), sharply increases the incidence of osteopenia and osteoporosis [21, 34, 35]. The PTH also has some moderating effects on osteoblasts and any imbalance in its concentration could result in bone loss and increased risk of fracture [36, 37].

3.1. Calcium-Mechanisms by which diabetes can affect the calcium metabolism are complicated, however in most parts they are related to calcium loss through kidney [38]. In diabetic rats, the kidney secretion of calcium is higher than non-diabetic rats. In addition the levels of 1,25(OH)2-D3 and vitamin D binding protein is almost ten times lower than normal group [21]. In diabetic mice there is an association between amount of calcium secreted in urine and decreased bone density [33]. The Quantified-PCR in the kidney showed changes in mRNA expression levels of 25-hydroxyvitamin D-24-hydroxylase (down regulation)and 25-hydroxyvitamin D-1α- hydroxylase(up regulation) as well as down regulation of calcium transferring receptors mRNA, plasma membrane Ca-ATPase and vitamin D receptors (VDR) genes [33].
3.2. Vitamin D- In the first two years of life, the vitamin D deficiency causes Rickets, while in adults vitamin D deficiency can induce or exacerbate osteoporosis or cause osteomalacia [39]. It has been shown that vitamin D has a role in pathogenesis of both types of diabetes [40]. In T1DM, it seems that vitamin D produces its effects through affecting the immune system, while in T2DM the ameliorating effects of vitamin D on B cell function and also increased insulin sensitivity is more prominent [41]. Moreover, T1DM might be associated to some genetic polymorphisms linked to vitamin D deficiency [42]. An association between vitamin D deficiency and diabetes incidence, management, complications, and mortality has been reported [43–45]. Although many studies indicate that vitamin D deficiency could be a risk factor for both types of diabetes [41, 46], there is still debate about whether vitamin D deficiency is a consequence of diabetes or a predisposing factor [47]. There are no randomized clinical trials investigate the effect of vitamin D supplementation on the incidence of diabetes.
3.3. PTH- Animal studies have shown that serum levels of PTH, vitamin D, calcium, Mg and Phosphate are lower in diabetic models than controls [48]. Moreover, in diabetic mice treatment with PTH-related protein, protein mostly produced by mature osteoblasts, can stop and even reverse the trabecular bone loss. This finding can indicate the modulatory and stimulatory effects of PTH on osteoblast function and maturation [36, 49, 50].

It has been shown that increased levels of PTH are associated with insulin resistance [51]. Moreover, there is an association between decreased levels of PTH and vertebral fracture in T2 diabetic patients [52], which could be related to attenuated anabolic effects of PTH on bones [37]. In young insulin-requiring diabetic patients serum levels of 1,25-(OH)2 vitamin D3 was decreased but PTH, calcium and Phosphate levels remained in normal range, probably with the cost of decreased cortical bone in these patients [35]. The anabolic effect of intermittent administration of PTH and its synthetic peptide fragment, teriparatide (PTH 1-34, TPTD) on bone has been well documented [53]. Increased serum levels of osteocalcin after PTH therapy may describe the anabolic mode of action [50].

There are trials demonstrated the bone healing effect of PTH and, a small number of case reports illustrated a potential role for TPTD to improve healing of nonunion and stress fractures in patients with diabetes [54, 55]. Moreover, the beneficial effects of PTH on osteoporosis caused by thiazolidinediones (TZDs) could be an interesting subject to be assessed by researchers [56].

4. Diabetic bone marrow adiposity

Recently, obesity is regarded as an inflammatory state that could result in metabolic disturbance [57]. The adipokines including leptin, adiponectin, visfatin and resistin have a role in insulin resistance in obese subjects and there is a significant relation between insulin resistance and obesity [57]. Bone fat has an important role in pathology of bone loss. Even recently a new term, osteopenic obesity, has been introduced that point out to effects of fat tissue on bone metabolism [58].

There are several animal studies in which a higher fat content in the bone marrow of diabetic subjects compared to their healthy counterparts are observed [49, 59, 60]. Adipocytes and osteoblasts have a common source, both originating from pluripotent mesenchymal stem cells (MSCs). MSCs can differentiate into these two cell lines; however the factors that affect this differentiation can also have effects on bone density. Adipocytes produce free fatty acids whose lipotoxic effects can directly inhibit the osteoblast proliferation [61, 62]. These fatty acids can result in an increased insulin resistance [63, 64].

To date, some of the factors affecting the differentiation of bone marrow cells have been known. For example the transcription factors runt-related transcription factor 2 (Runx2) stimulates the osteogenesis and peroxisome proliferator-activated receptor γ (PPARγ) stimulates the adipogenesis [65]. Hyperglycemia can stimulate the differentiation of MSCs to fat cells [66–68]. For example in tibia of type 1 diabetic rats, the expression of PPARγ as well as the number of fat cells is increased when compared to control [64]. BMD and trabecular volume is also increased in mouse with birth defect in PPARγ [69]. Some anti-diabetic medications may induce osteopenia with same mechanism. For example in mice, rosiglitazone therapy, as the agonist of PPARγ [65], can result in a massive bone loss as well as increase in the fat content of bone [69]. However it cannot cover for all the osteopathies seen in diabetes. In diabetic rats, therapy with antagonists of PPARγ decreases the fat content of bone marrow; however it cannot inhibit the bone loss and attenuation of BMD. This observation indicates that only part of bone loss seen in diabetes is related to PPARγ mechanism and there is still so many areas in this matter that needs to be assessed [59].

5. Secretion of bone marrow cells
  5.1. Adipocyte-derived hormones- Adiponectin, Leptin, Resistin

In-vitro as well as animal studies is has been shown that adipose-derived hormones (such as adiponectin, leptin, visfatin, and resistin) have role in bone metabolism [70], however their complete mechanism of action is not clear. Therefore, it is not reasonable to employ the adiponectin as a bone health assessment factor [71].

5.1.1. Adiponectin

Adiponectin is a hormone secreted by fat tissue with anti -inflammatory effects. This hormone can increase the insulin sensitivity and also regulates the energy hemostasis [72]. Their receptors are located on osteoblast which might indicate the role of this hormone in fat and bone metabolism [73]. Its effects are not clearly understood, although some of its known actions on bone metabolism seem to be opposite. For example in adiponectin knock out rats, a decrease in trabecular bone and an increase in bone marrow fat are observed. Beside, adiponectin treatment in these rats decreases the osteoclast number and increases the trabecular bone. While this treatment can increase the conversion of stromal cell of bone to osteoblasts [74], at the same time can activate the receptor activator of nuclear factor kappa-b ligand (RANKL) which has negative effects on bone metabolism [51].

In clinical studies an inverse relation between adiponectin levels and BMD is observed, which is less prominent in obese subjects and increases with weight loss [75]. Women has higher levels of serum adiponectin compared to men [75]. There is also some evidence that indicate the increased insulin sensitivity caused by adiponectin is limited to female subjects [76, 77]. Furthermore, it has been shown that increased levels of adiponectin and not leptin, can independently increase the risk of fractures [70]. The altered levels of adiponectin is reported in various disease [75]. In osteoarthritis the adiponectin secretion is decreased [75]. in obese subjects the low serum levels of adiponectin showed an association with T2 DM, metabolic syndrome, atherosclerosis and cardiovascular disease [78]. Although the decreased levels of adiponectin in T2 diabetic subjects are associated with increased risk of cardiovascular disease [57], the levels of adiponectin are increased in T1 diabetic patients who suffer from micro vascular complications [79]. However it is not clear that this increase reflects the pathological role of adiponectin in microvascular complications of diabetes or is a beneficial response due to counter-regulatory effects. Investigating the association between this hormone and various diseases, another potential affecting factor, except gender, which worth to be considered is BMI. This could lead to obtain a better understanding of the relationships between variables and also using these relationships in the process of predicting the occurrence of diseases.

Either way full understanding of adiponectin relevant interventions not only can be useful in understanding the mechanisms of diabetic osteopathy but also might help discover the new therapies for diseases such as diabetes, metabolic syndrome, cardiovascular disease, and obesity.

5.1.2. Leptin

Leptin has a regulatory role in bone metabolism. This protein by affecting the stromal cell of bone marrow inhibits the differentiation toward osteoclast and stimulates the osteoblast production [80–82]. In T1 diabetic mice, leptin levels is significantly low with increased bone marrow fat and decrease bone mass [80]. Administration of leptin to leptin-deficient ob/ob non- diabetic fatty mice deceases the fat content of bone and increase the bone mass [83]. On the other hand, administration of leptin to T1 diabetic mice decreases the bone fat, however it cannot inhibit the bone loss caused by diabetes [83]. In clinical studies, the results are conflicting. T1 diabetic subjects are presented with elevated [84] or very little attenuated levels of leptin [85]. The dissimilarities might be contributed to low BMI in this group of patients (T1DM). In T2 diabetic subjects BMI is usually higher and therefore the serum concentration of leptin is increased. However, it has been proven that leptin levels, independently of BMI, has a positive association with BMD [86]. In diabetic Hispanics individuals, a significant inverse relationship has been shown between serum leptin and BMD as well [87]. Similar to adiponectin and IGF-1, it seems understanding the mechanisms by which leptin produces its effects on bone and fat tissues can lead to development of new therapeutic strategies in the treatment of various diseases.

5.1.3. Visfatin and resistin

Similar to adiponectin, visfatin can increase the insulin sensitivity [57]. The visfatin, highly expressed by cells of bone marrow, produces its effects on bone metabolism through affecting the osteoblasts; increasing the glucose absorption and mRNA levels of osteogenic factors [88]. Visfatin levels are higher in obese subjects [57]. A meta-analysis showed that visfatin can be used for prediction of risk of insulin resistance, diabetes, and metabolic syndrome [89].

In contrast to visfatin that has insulin-like effects, resistin has anti-insulin effects, an effect that stands for its name (resistin for resistance to insulin). Anti-diabetic agents such as pioglitazone can decrease its concentration in serum [63]. In leptin knock out ob/ob obese mice, increased levels of resistin are observed which indicate the association between resistin and leptin [90].

5.2. Osteoblast derived hormones: Osteocalcin

Osteocalcin (OC), as the major protein secreted by osteoblasts, is involved in glucose metabolism. OC mediates the insulin secretion from pancreas and also has a positive effect on adiponectin secretion from fat cells [91]. OC even in a very low concentration (at picomolar range) can be sufficient for expression of insulin genes and B cell proliferation markers [92].

In mice lacking the OC, decreased B cells proliferation, insulin resistance, and abnormal levels of visceral fat is reported [91]. In male mice, OC increases the testosterone levels. It has been hypothesized that the effects of OC on metabolism is mediated through testosterone as well [76]. The last but not the list, The OC levels have positive correlation with adiponectin and it is likely that combination of these two proteins produces some positive effects on insulin sensitivity [93].

In accordance to animal studies, serum levels of OC and/or undercarboxylated osteocalcin (ucOC) are shown to be decreased in patients with diabetes (T1DM and T2DM) [94, 95]. Furthermore, it seems there is an inverse association between serum levels of OC and HbA1c; along with better control of diabetes, serum levels of OC increase [95]. Although, some studies are done which do not confirm this association.[96, 97]. Despite the fact that taking alendronate would cause decrease in the OC and ucOC levels, two recently published cohort studies have shown that alendronate might reduce the risk of developing Type 2 diabetes [96, 97]. One explanation for this paradox could be the not exactly identified role of ucOC in human glucose metabolism [98].

6. Inflammation

There is evidence point to the role of inflammation in development of diabetes. It is included chronic systemic inflammation as well as bone inflammation. In animal models, it has been shown that chronic inflammation can result in osteoporosis [99]. In clinical studies, an association between osteoporosis and inflammatory bowel syndrome is reported [99]. On the other hand, activation of immune system has a role in both types of diabetes although its role is more prominent in T1 DM [100, 101]. We have the autoimmune destruction of B cell in T1DM, while the inflammatory processes in T2DM are more chronic low-grade inflammatory process [100, 101].

Bone inflammation is one of the factors by which onset of type 1 diabetes is marked, identifying the role of pro-inflammatory cytokines present at the early stages of diabetes onset will provide an essential information about the cause of diabetic bone loss as well as new potential protective treatment [102]. Evidence supporting this hypothesis is as follows: experimental studies performed on T1DM mice showed that in early days following diabetes induction, local and systemic inflammatory cytokines are increased [59, 103],Simultaneously with this process, the markers of osteoblast inhibition as well as adipocytes markers are increased [102, 103].

7. Vasculopathy

Microangiopathy is another mechanism that is thought to result in bone loss in diabetes [104]. In brain of diabetic mice, decrease in brain perfusion as well as three times attenuation of cumulative vascular density is noted compared to control group [105]. In 1981, a research performed on 118 diabetic subjects revealed that in 82 % of iliac crest biopsy, patients showed diabetic microangiopathy, prominent osteopenia and decreased sinusoidal capillaries [106]. Although this research could not be repeated because of ethical issues, later findings strengthened the hypothesis that diabetic microangiopathy is one of the causes of diabetic osteopenia. In 1983, Demmler et al also showed there was a decline in density of arterial capillaries in the osteoporotic bone [107]. It has been shown that macrovascular and microvascular diabetic complications can be indirectly linked to increased fragility fracture risk, considering fall frequency as a confounder [104]. In a study done by Burkhardt and colleagues, no significant association was found between diabetic angiopathy and BMD, however in T1DM women with coronary heart disease (CHD) a trend for lower femoral neck BMD than those without CHD was reported. These findings support the above hypothesis [108].

8. Anti-diabetic agents

Insulin has some anabolic effects on bone [109]. On the other hand, in patients inject insulin the occurrence of hypoglycemia increases the fall-related fractures, there is a direct association between daily insulin need and low BMD. This might be due to the fact that higher daily insulin requirement is an indicative of disease progression and complications [110].

Oral anti-diabetic agents produce various and sometimes opposite effects on bone metabolism.

8.1. Agents with positive effects on bone metabolism
  8.1.1. Metformin and sulfonylurea have protective effects on bone fractures [111]. Both agents showed osteogenic effect in in-vitro studies that is mediated through increased proliferation and differentiation toward osteoblasts [111].
  8.1.2. Dipeptidyl peptidase-4 (DPP-4) inhibitors such as sitagliptin has anabolic effects on bone [112].
8.2. Agents with negative effects on bone metabolism

TZDs including pioglitazone and rosiglitazone decrease the differentiation of bone marrow cell toward osteoblast and increase the adipocyte differentiation [113]. A meta-analysis showed that consumption of this group of anti-hyperglycemic agents is associated with increased risk of fracture specially in women [56] . More recently it was suggested that some clinical trials should be performed in order to assess the effects of PTH therapy in prevention of fracture in patients consuming these agents [56].

It should be noted that long term effects of anti-diabetic medications on bone metabolism are missing and to have a broad and accurate conclusion such studies are required.

## Conclusions

There is a lot of evidence that demonstrate both types of diabetes have a negative effect on bone strength and both types are associated with an increased risk of fracture [4, 5, 108]. We might suggest that among mechanisms explored in this paper, the hyperglycemia and AGEs have more prominent role [9]. However, studies that focus on other mechanisms by which diabetes affect the fragility of bones are highly recommended. In this way we are more likely to find practical therapeutic approaches for proper and timely management of osteoporosis.

Evaluation of effects of diabetic complications such as micro- and macro-angiopathy, nephropathy and neuropathy on bone health can also be helpful. Bone itself can affect the glucose metabolism and diabetes risk [91, 95]; however, the underlying mechanism by which the bone affects the metabolism of the body is still elusive. In this regard, another point is to stress on conducting studies related to bone-derived hormones. All debates that we mentioned above can express a new interesting concept. Based on this concept, bone is not a simple organ that only gets influence from other organs, but it is an endocrine organ that by secreting the agents such as osteocalcin, adiponectin, and visfatin can affect the body metabolism.

## Abbreviations

BMD: Bone marrow density
BTMs: Bone turnover markers
CHD: Coronary heart disease
DPP-4: Dipeptidyl peptidase-4
IGF-1R: Insulin-like growth factor type 1 receptor
MSCs: Mesenchymal stem cells
OC: Osteocalcin
PPARγ: Peroxisome proliferator-activated receptor γ
RANKL: Receptor activator of nuclear factor kappa-b ligand
Runx2: Runt-related transcription factor 2
T1DM: Type 1 diabetes
T2DM: Type 2 Diabetes
TZDs: Thiazolidinediones
ucOC: Undercarboxylated osteocalcin
